# CD44 and its ligand hyaluronan as potential biomarkers in malignant pleural mesothelioma: evidence and perspectives

**DOI:** 10.1186/s12931-017-0546-5

**Published:** 2017-04-12

**Authors:** Lourdes Cortes-Dericks, Ralph Alexander Schmid

**Affiliations:** Department of Clinical Research, Division of General Thoracic Surgery, University Hospital Berne, Berne, Switzerland

**Keywords:** CD44, Hyaluronan, Malignant pleural mesothelioma, Biomarker

## Abstract

Malignant pleural mesothelioma (MPM) is a rare and highly drug resistant tumor arising from the mesothelial surfaces of the lung pleura. The standard method to confirm MPM is the tedious, time-consuming cytological examination of cancer biopsy. Biomarkers that are detectable in pleural effusion or patient serum are reasonable options to provide a faster and noninvasive diagnostic approach. As yet, the current biomarkers for MPM lack specificity and sensitivity to discriminate this neoplasm from other lung tumors. CD44, a multifunctional surface receptor has been implicated in tumor progression in different cancers including MPM. The interaction of CD44 with its ligand, hyaluronan (HA) has demonstrated an important role in modulating cell proliferation and invasiveness in MPM. In particular, the high expression levels of these molecules have shown diagnostic relevance in MPM. This review will summarize the biology and diagnostic implication of CD44 and HA as well as the interaction of both molecules in MPM that will demonstrate their potential as biomarkers. Augmentation of the current markers in MPM may lead to an earlier diagnosis and management of this disease.

## Background

MPM is an aggressive, and locally invasive tumor emanating from the mesothelial cells of the pleura mainly due to chronic exposure to asbestos fibers [1–3]. Although it is well accepted that asbestos is the major causative agent of MPM, the number of cases involving persons with less asbestos exposure is also increasing [4]. The diagnosis of MPM is complex because of its composite epithelial/mesenchymal patterns, its phenotypic variability from patient to patient, and its property to mimic other cancers particularly, adenocarcinoma or benign processes [5]. Histologically, MPM is divided into epitheloid, sarcomatoid and biphasic subtypes, which has an impact on diagnosis, treatment and prognosis of the disease. The epitheloid is the most common and least aggressive representing 50–70% of all MPM cases. The sarcomatoid, a less prevalent subtype is the most aggressive form and by far difficult to manage. The biphasic, a combination of both epitheloid and sarcomatoid comprises 20–35% of all MPM cases [6, 7]. MPM is highly tolerant to all standard treatments that include the first-line chemotherapy consisting of cisplatin and pemetrexed, surgery and radiation [1]. Response rate to standard chemotherapy is 20–40% with a median survival time of less than 12 months [6, 8]. Prognosis remains poor because of the difficulties of early diagnosis, hence, application of biomakers with high sensitivity particularly for the early stage of the disease remains a continuing task [9, 10].

The National Cancer Institute defines a biomarker as a biological molecule found in the blood, body fluids or tissues representing a sign of a normal or abnormal process, or of a condition or disease. Biomarkers include proteins (e.g. enzyme or receptor), nucleic acids (e.g. micro RNA), antibodies and peptides and, thus, can be easily obtained noninvasively and serially [11], and neither require biopsy or special imaging for evaluation [12].

One of the established tumor biomarkers is the cluster of differentiation 44 (CD44), an adhesion/homing molecule and the major receptor for HA [13–15]. As a multifunctional cell surface receptor, CD44 has been associated in the development of many neoplasms because of its modulating ability in cancer progression such as conveying cell adhesion and cell migration that aids the expansion of tumors [16–18]. CD44 has also been dubbed as a putative cancer stem cell (CSC) in lung cancers and MPM because of its significant influence on disease progression and negative treatment outcome [19–25]. These attributes render CD44 as a biomarker in screening, differential diagnosis and prediction of response to therapy [26]. HA, the most common ligand of CD44 [19] is a glycosaminoglycan (GAG), which is widely distributed within the extracellular matrix. In this compartment, HA regulates different cellular activities such as cell migration, growth and differentiation and cell adhesion [27–30]. High levels of HA in pleural effusions of MPM patients have shown evidence of its diagnostic value in MPM sustaining the idea of being a predictive biomarker in this tumor [31–37]. Herein, we will describe the biology of CD44 and HA, and summarize their diagnostic performance in MPM. It will also discuss the inaccuracy of the conventional biomarkers in pleural fluids/serum for MPM as well as the rationale why CD44 and HA may serve as diagnostic biomarkers that may add to an earlier diagnosis and commencement of appropriate therapies in this disease.

## CD44 molecule

CD44 is a cell surface adhesion molecule involved in cell-cell and cell-matrix interactions [16, 38]. The interaction of CD44 with its ligand and associated molecules regulate cell adhesiveness, cell motility, matrix degradation, cell proliferation and survival that potentiate its crucial role in carcinogenesis [17, 18]. CD44, the major receptor for the hyaluronan (previously named hyaluronic acid, HA) is endogeneously expressed at low levels in different types of normal tissues that necessitates activation before binding to HA [39, 40]. Variant isoforms of CD44 specially, CD44 v6-v10 are overexpressed in both human and animal neoplasms indicating its implication in cancer progression, whereas its removal is associated with inhibition of tumor growth [41–44]. Inhibitors such as exemestane, trametinib and statin exert profound antiproliferative effects on mesothelioma growth either by a direct downregulation of CD44 [45] or the suppression of CD44 and its associated signaling pathways [46, 47] attesting the critical role of CD44 in modulating tumor growth in MPM.

The CD44 structure on normal cells is different from that on tumor cells because under various physiological and pathological conditions, the local environmental pressure alters splicing and post-translational modifications to produce various types of CD44 molecules with enhanced HA binding that triggers increased tumorigenicity [13, 16, 48–51]. CD44 can be cleaved at the membrane-proximal region of the ectodomain by MT1-MMP (membrane type 1 matrix metalloproteinase), which is thought to play an essential role in CD44-mediated tumor cell migration alongside with extracellular matrix components [52–54]. Although all CD44 isoforms are endowed with HA recognition sites, not all cells bearing CD44 bind the HA ligand constitutively. In addition to HA, CD44 interacts with different ECM proteins such as fibronectin, collagens, growth factors, cytokines, chemokines, matrix metalloproteinases and osteopontin [16].

**Fig. 1:**
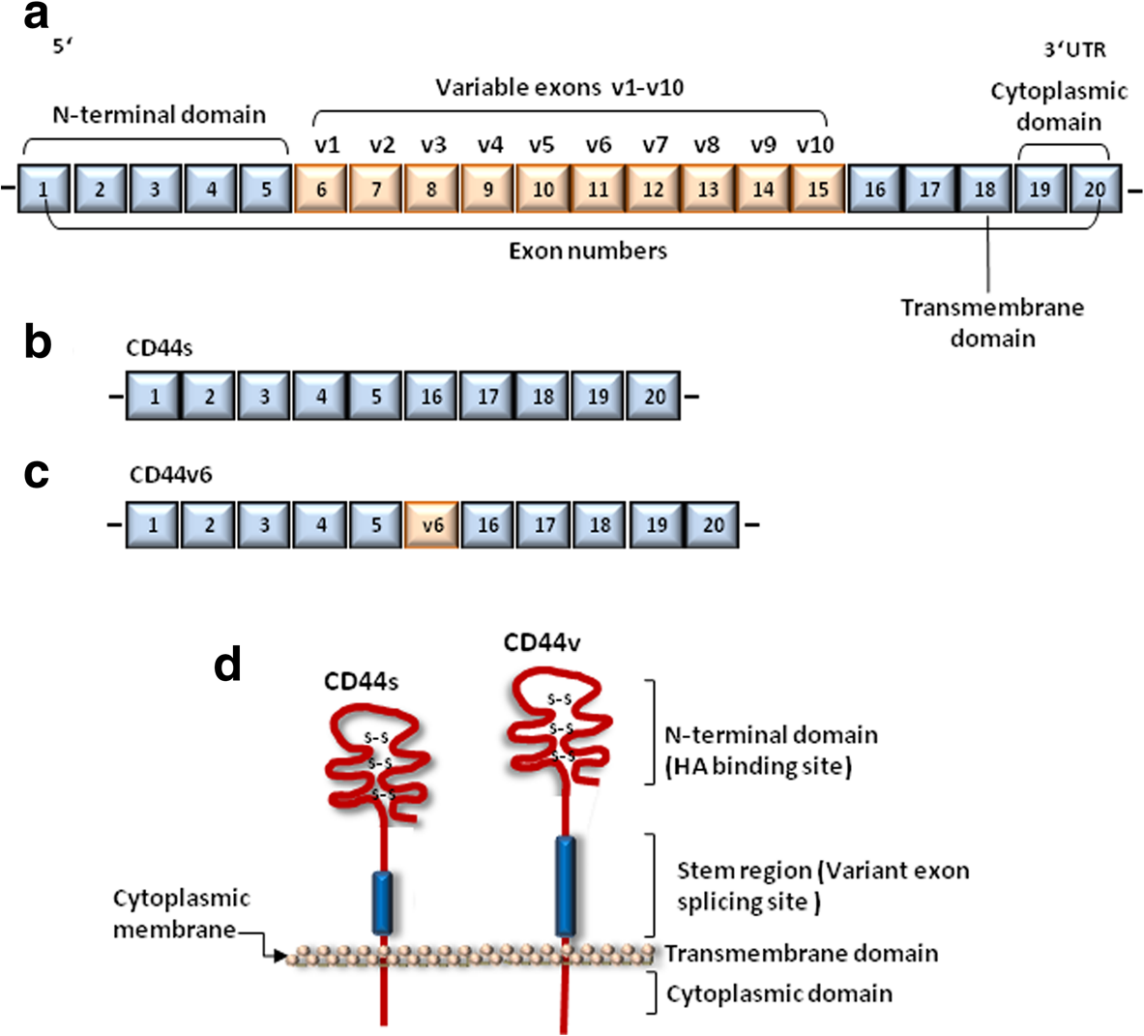
Genomic and protein structures of CD44. **a** The human CD44 gene consists of 20 exons. Those encoding for N-terminal, transmembrane and cytoplasmic domains as well as the exon numbers are shown. Alternative splicing of variable exons, v1-v10 generates CD44 variant isoforms. **b** The standard CD44 (CD44s) consists of exons 1–5, 16–20. **c** The variant form, CD44v6 contains the standard exons 1–5, 16–20 and variant exon 11 (v6). **d** CD44 is a single-pass transmembrane glycoprotein consisting of four functional domains: N-terminal domain, stem region, transmembrane domain and the intracellular cytoplasmic domain. N-terminal domain, the site for HA binding is bound together by three cysteine disulfide cross-links. Alternative splicing occurs at the stem region generating the variant form. The transmembrane domain anchors the molecule in the cytoplasmic membrane, whereas the cytoplasmic region conveys essential functions of CD44. v, variant exon, UTR, untranslated region

Twenty exons are involved in the genomic organization of CD44. The first five and the last five exons are constant whereas the ten exons located between these regions are subjected to alternative splicing from which the variable regions emanate (Fig. 1a). The smallest molecule (85–95 kDa) without the variable region is the standard CD44 (CD44s) (Fig. 1b). As this is found mainly on cells of lymphohematopoietic origin, CD44s is also known as hematopoietic CD44 (CD44H). After immunological activation, T lymphocytes and other leukocytes transiently upregulate CD44 isoforms expressing variant exons designated as CD44v. A CD44 isoform containing the last exon products of the variable region, CD44v8-10 is also known as epithelial CD44 (CD44E) and is preferentially expressed on epithelial cells [50]. CD44v6 (CD44 variant exon 6) is the major CD44 isoform that regulates tumor invasion, progression and metastasis [18, 55] (Fig. 1c). Several MPM cell lines are positive for CD44v9 (variable exon 9) including the CD44v8-10, the former being statistically associated with NF2 (neurofibromatosis type 2), a common feature of MPM [56]. The protein structure of CD44 consists of an N-terminal HA-binding link-homology motif, stem region, transmembrane domain and short C-terminal cytoplasmic region (Fig. 1d). CD44 binds to its major ligand, HA via the N-terminal HA- binding link-homology motif. The C-terminal cytoplasmic region has a major role in eliciting the essential functions of CD44 in the regulation of intracellular signal transduction through binding to different molecules such as the cytoskeleton components, kinases and activators of small Rho GTPases [57, 58].

## CD44 ligand, hyaluronan

Balazs et al. [59] introduced the term “hyaluronan” in 1986 to conform with the international nomenclature of polysaccharides to include the different forms this molecule can take such as the acid form, hyaluronic, and the salt form, sodium hyaluronate. [60]. HA is a non-sulfated, linear GAG consisting of repeating disaccharides of (ß, 1–4)-glucoronic acid (GlcUA) and (ß, 1–3)-N-acetyl glucosamine (GlcNAc). HA is synthesized at the cell plasma membrane by specific hyaluronan synthases (HAS); HAS-1, HAS-2 and HAS-3 and is directly released into the extracellular matrix [14, 30]. At the cellular level, HA plays essential roles in modulation of tissue architecture, cell motility, cell proliferation and is a prominent component of the microenvironment in most malignant tumors [14, 42]. The diverse functions of HA within the extracellular matrix is a result of the different HA-binding receptors such as CD44, RHAMM (receptor for HA-mediated motility), other receptors bearing HA-binding motifs such as the transmembrane protein layilin, HARE (HA receptor for endocytosis), LYVE-1 (lymphatic vessel endocytic receptor), intracellular HA-binding proteins including CD37, RHAMM/IHABP (intracellular HA-binding protein), P-32 and IHABP4 [28, 60].

Upregulated HA production is supposed to produce less dense matrix, hence, providing the cell a suitable platform for increased cell motility and invasion property [61]. Because HA not only provides a cellular support and hydrophilic matrix but also facilitates cell-cell adhesion, cell migration, growth and differentiation, these properties bestow HA as a suitable candidate in modulating pathological processes such as cancer [29, 62]. Elevated levels of HA have been detected in different types of human cancer and its accumulation within the tumor stroma has been associated with poor prognosis and survival in cancer patient [63]. In liver fibrosis, increasing levels of serum HA have been measured during the progression of the disease [64–66].

**Fig. 2:**
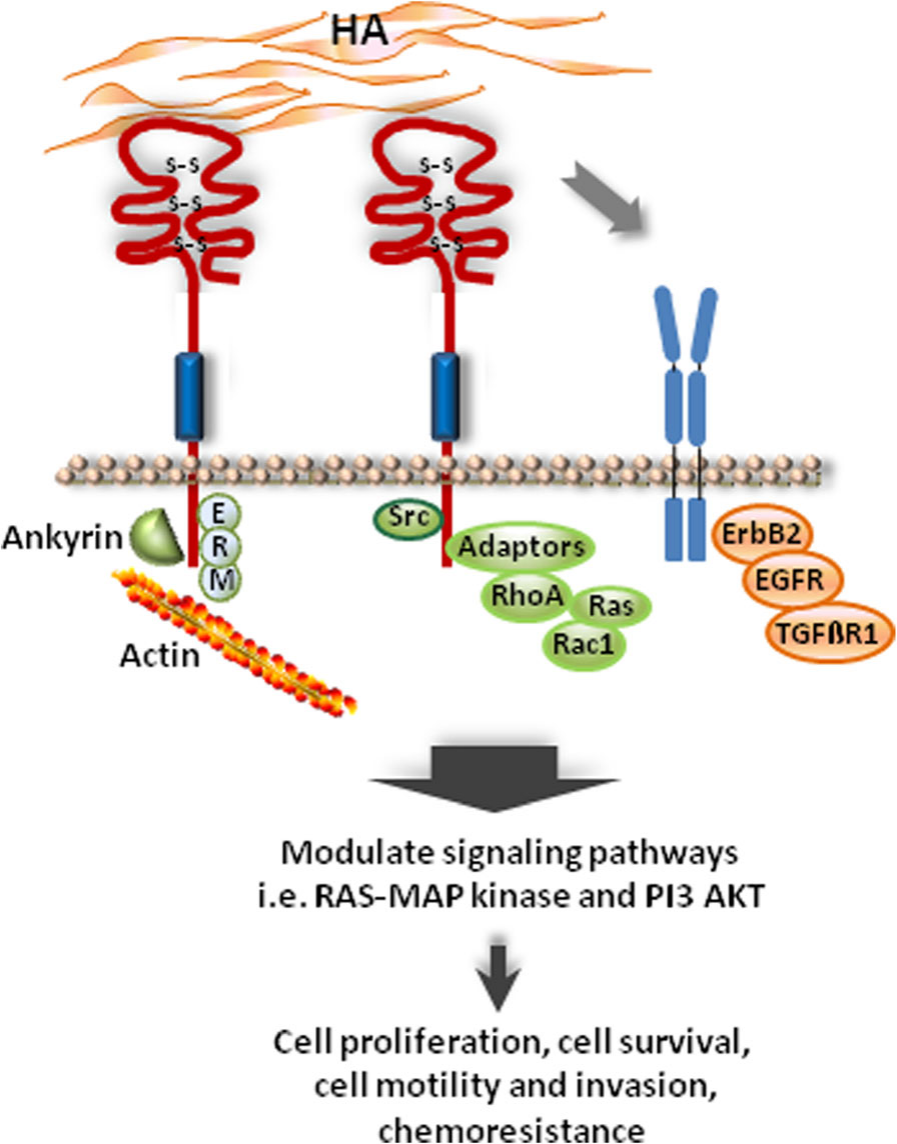
CD44/HA interaction in tumor. CD44/HA binding in the malignant setting elicits an interaction of CD44 with ErbB2, EGFR and TGFßR1 that alter the physiological effects of these receptors. It transmits signals through the cytoplasmic domain after binding with diverse cytoskeletal proteins such as actin-cytoskeleton adaptor protein, ankyrin, ezrin-redixin-moesin (ERM) proteins, intracellular signaling components including the Rho family GTPases, Src kinases and associated molecules. To a great extent, MAP kinase and PI3 kinase/AKT signaling mediate cell proliferation, cell survival, cell motility and invasion as well chemoresistance [14]. ErbB2, epidermal growth factor receptor-2, EGFR, epidermal growth factor receptor, TGFßR1, transforming growth factor beta receptor 1, GTPases, family of hydrolase enzymes that bind and hydrolyse guanosine triphosphate (GTP)

In tumors, HA binding to CD44 evokes an interaction of CD44 with signaling receptors such as the epidermal growth factor receptor-2 (ErbB2), epidermal growth factor receptor (EGFR) and transforming growth factor beta receptor type 1 (TGFßR1) that consequently alters the physiological effects of these receptors [67–69]. It can also interact and consequently modify the activity of nonreceptor kinases of the Src family or Ras family GTPases, and with switch molecules such as RhoA, Rac1 and Ras via adaptor proteins that generate intracellular signaling circuits [14, 15, 67, 68, 70, 71]. In such a way, CD44/HA binding modifies the activity of different downstream signaling cascades, in particular, the MAP kinase and PI3/Akt pathways and consequently convey tumor cell proliferation, cell survival, cell motility and invasiveness and chemoresistance [14, 72] (Fig. 2).

## CD44/HA interactions in MPM

CD44 in cancer cells interacts with hyaluronan-rich microenvironments modifying cell signaling pathways that trigger the ability of malignant cells to migrate, to invade basement membranes and to lodge at distant sites of the tumor [14, 70, 73]. That CD44/HA binding mediates several tumor cell-specific activities and progression indicates that CD44/HA interactions play a pivotal role in cancer development [67, 71]. In MPM, the presence of HA and its receptor, CD44 have been shown to facilitate neoplastic cell motility and invasion linking CD44/HA interactions in tumor progression.

Li and Heldin [27] provided evidence that the overexpression of hyaluronan synthase 2 (HAS2) in a nonhyaluronan producing MPM cell line, Mero-25, changed the histological type of Mero-25 from less aggressive epitheloid to a more aggressive fibroblastic phenotype. These authors further observed that HAS2-transfected cells demonstrated a two-fold increase in the expression of the HA receptor, CD44 accompanied by increased cell motility, thus, disclosing the critical role of HA in the dissemination of mesothelioma cells in adjacent nontumor tissues. In a separate study, HA/CD44 interactions in the 2 MPM cell lines ACC-MESO-1 and K921MSO, exhibited a crucial role in driving cell proliferation and invasiveness. These MPM cell lines, which express high quantities of CD44 demonstrated enhanced cell proliferation and upregulated invasion capacity after HA treatments, in vitro. Notably, it was found that silencing of CD44 significantly abrogated the HA-mediated cellular effects in the 2 MPM cell lines indicating the essential role of CD44/HA binding in modulating migration and proliferation of mesothelioma cells [74].

A comparative analysis between the low molecular weight hyaluronan (LMWHA) and high molecular weight hyaluronan (HMWHA) has been performed to assess their capacity in mediating cell proliferation and migration on 4 MPM cell lines, expressing CD44. This study made evident that the MPM cell line expressing the highest level of CD44 was induced by LMWHA, which resulted to an increased cell proliferation and haptotaxic migration. In this work, CD44 neutralization profoundly reduced LMWHA binding to MMC (malignant mesothelima cells) illustrating that the interaction between CD44 and HA components transmit regulatory signals conferring locomotion and proliferation of MMC and, thus, show their participation in the expansion of tumor [7].

A mechanistic CD44/HA association in MPM has been observed by Asplund and Heldin [75]. These authors found HA-binding sites on the cell surface of three tumor-derived MPM cell lines, which were absent in normal mesothelial cells. The HA receptors appeared to be related to CD44 because the human CD44 monoclonal antibody, Hermes-1, which prevented the binding of HA to CD44 suppressed the major part of the HA binding mechanism. However, no strict correlation was recorded between the HA binding activity on the tested cells, and the levels of CD44 molecules on their cell surfaces indicating that only a fraction of the HA was bound to the CD44, and that other HA binding proteins may also exist. This study also revealed that the pleural fluids from MPM patients were capable of stimulating HA synthesis in primary mesothelial cultures expressing functionally active HA receptors, CD44.

Osteopontin (OPN), a secreted tumor-associated, noncollagenous phosphoprotein is an extracellular matrix component and a cytokine through binding to its receptors integrin and CD44 [76, 77]. An important role of OPN in MPM has been reported such as the modulation of multidrug resistance via osteopontin-dependent regulation of HA-CD44 interaction. Tajima and coworkers [78] presented evidence that the insertion of OPN gene in stable transfected ACC-MESO-1/OPN cell line upregulated the multidrug resistance through the mechanism of enhanced HA binding. Notably, the inhibition of HA-CD44 interaction abrogated multidrug resistance in the ACC-MESO-1/OPN sustaining an important role of HA-CD44 association in the regulation of drug tolerance and, thus, demonstrate that OPN is essentially implicated in conveying multidrug resistance by enhancing CD44 binding to HA.

## Diagnostic relevance of CD44 in MPM

As in the case of other biomarkers, the utility of CD44 as a diagnostic and/or prognostic marker was originally suggested because of its altered overexpression in human tumor tissues as compared with the nontumor or corresponding normal tissues. An early study of Penno et al. [79] has demonstrated that CD44 can be detected immunohistochemically in MPM tissues illustrating the presence of neoplastic cells. In this project, using a CD44 monoclonal antibody (H4C4), 92% of the MPM samples were found to contain 50–100% CD44 relative to the total number of cells. It was further noted that CD44 expression was related to the histological type of MPM, with the highest levels observed in the epitheloid subtype and the least in sarcomatoid. These authors also elucidated that increased CD44 levels modulates the association with HA, a major component of the pleural fluid.

Because MPM is more aggressive than pulmonary adenocarcinoma (ADC), Attanoos and coworkers [80] tested the diagnostic potential of CD44 to discriminate the two neoplasms by immunohistochemistry (IHC) in reactive mesothelium, pleural mesothelioma and pulmonary ADC. A strong immunoreactivity of CD44H (CD44s) was detected in all 20 biopsies of reactive mesothelium and in 75% of MPM specimens as compared to only 15% in pulmonary ADC, thus, proposing the utility of CD44H as a positive mesothelial marker alongside with established immunohistochemical indicators to differentiate ADC from MPM. CD44 localization in addition to HA by IHC has also been reported to discriminate MPM from ADC [81]. Membranous staining for CD44s was assessed positive in 100% of mesothelial hyperplasia, 86% of malignant mesothelioma and 8% of lung ADCs indicating that CD44s is a reliable marker to distinguish MPM from ADC. In yet another study, low CD44 expression was detected in MPM (57.7%) and in mesothelial hyperplasia (11.5%) using immunohistochemical analysis. Despite of the low expression of CD44 in the malignant samples, it was suggested that the mere presence of CD44 confirmed its potential as a positive mesothelial marker in the differential diagnosis of pleural neoplastic proliferation [82].

Using CD44, hyaluronan and HASs as putative markers for differentiating MPM from adenocarcinomas (ADCs), Törrönen et al. [83] recently found a surprisingly low CD44 positivity of stromal cells in ADCs as compared to mesotheliomas (MPMs). This report is unanticipated in the sense that, most results have accounted for an increased CD44 levels in MPM in contrast to those in ADCs [79, 80].

Porcel and colleagues [84] also compared the diagnostic utility of the concentrations of CD44v6 and CD44s in pleural fluids as biomarkers for distinguishing MPM from nonmesothelioma tumors. The CD44v6/CD44s ratio obtained from the concentration levels between MPM and other pleural malignancies has been proven as a reliable diagnostic tool for the differential diagnosis of malignant pleural effusions such that those with a high probability of being metastatic and, hence, preventing the need of an invasive thoracoscopy. An overview of the diagnostic relevance of CD44 in MPM is shown in Table 1.

**Table 1:** Diagnostic relevance of CD44 in MPM

Function	Method/specimen	Reference
High CD44 level indicates presence of neoplastic cells	IHC/MPM tissues	Penno et al. 1995 [79]
High CD44 level differentiates MPM from ADC	IHC/reactive mesothelium, MPM, ADC samples	Attanoos et al. 1997 [80]
CD44/HA localization discriminates MPM from ADC	IHC/paraffin-embedded serous fluids	Afify et al. 2005 [81]
CD44v6/CD44s ratio aids in differential diagnosis between MPM and non-MPM tumors	ELISA/Pleural fluids	Porcel et al. 2011 [84]
Low CD44 level aids in differential diagnosis of pleural neoplastic differentiation	IHC/epitheloid MPM and reactive mesothelial hyperplasia tissues	Ali et al. 2013 [82]
Low CD44 positivity and high HA staining may differentiate MPM from ADC	IHC/MPM tissues	Törronen et al. 2016 [83]

*MPM* malignant pleural mesothelioma, *ADC* adenocarcinoma, *HA* hyaluronic acid, *IHC* immunohistochemistry, *ELISA* enzyme-linked immunosorbent assay

## Diagnostic value of HA in MPM

One of the earliest pathological reports on HA was its isolation in 1939 from the pleural fluid of a patient with a malignant tumor of the pleural and peritoneum, which demonstrated that neoplastic cells secrete HA [85]. This was followed by a consensus that the high concentration of HA in the pleural effusions or ascites of MPM patients is a standard finding [37, 86, 87] or is associated with MPM [83]. Indeed about 70% of MPM patients exhibit high levels of HA in pleural effusions or serum [88] in which a direct correlation has been found between the upregulated hyaluronan levels in the circulation and tumor damage in HA-producing mesotheliomas [34, 35, 88].

One parameter by which MPM affirms its mesenchymal origin is by the formation of HA, making it as one of the most important criteria in distinguishing between MPM and metastatic ADC [80, 87, 89]. In fact, tumor-secreted HA in the pleural fluid has been suggested as a means of identifying MPMs [31, 33, 81, 86, 90–93]. In this context, Waxler and coworkers [94] developed a method for isolating HA and other GAGs from tumor tissues and observed that MPMs contained only or almost entirely HA, whereas carcinomas and sarcomas consisted of a mixture of HA and other GAGs. Based on this, these authors concluded HA as the sole or major GAG to confirm a diagnosis of MPM. Another study suggested that increased total GAG aids in the differential diagnosis between MPM and diffuse ADC [95] and is a basic finding in addition to the elevated levels of HA and chondroitin sulfate in MPM [96]. Welker et al. [97] further recommended that the combination of HA and cytology may even improve the diagnosis of MPM.

An earlier report has found that HA values of > 0.25 μg/ml in pleural effusions indicates the presence of MPM [86]. Using a higher cut-off level of 100 μg/ml for HA, Atagi et al. [92] as well as Petterson and colleagues [33] reported that such a high concentration of HA in the pleural fluid combined with a low concentration of carcinoembryonic antigen (CEA) aid in the differential diagnosis for MPM. As a single marker, HA values of >100 μg/ml has been recommended as a diagnostic indicator for MPM [31].

High serum levels of HA in MPM patients have been measured in MPM patients in later and progressive stages [34] denoting HA as a marker of a progressive disease [35, 88]. An accumulation of high intracellular HA, a feature that is not reported in ADC, could distinguish MPM from ADC according to Afify and coworkers [81]. Their study revealed that all MPMs and 93% of the benign mesothelial cells were positive for intracytoplasmic HA as compared with a 100% negativity in ADCs. In contrast to this report, the group of Chiu [87] claimed that HA is neither the sole nor the predominant GAG in most MPMs. These authors found that quantitatively, MPMs exhibited statistically higher amounts of HA than primary lung ADCs but were not statistically different from soft tissue sarcomas or primary ovarian serous neoplasms. Hence, they suggested that high levels of HA support the diagnosis of MPM when the alternative diagnosis is primary ADC of the lung. Intriguingly, high levels of HA in pleural fluid have been proposed not to be specific for MPM as it can also occur in other malignant or benign diseases and a low level does not exclude MPM [98].

Another aspect of importance is that the pleural fluids from MPM patients exhibited profound HA-stimulatory activity as compared with the nonmesothelioma fluids, thus, demonstrating that HA-binding capacity may serve as an additional marker in combination with other diagnostic tools to delineate between MPM and normal mesothelial cells [99]. Table 2 shows an overview of the diagnostic value of HA in MPM.

**Table 2:** Diagnostic value of HA in MPM

Function	Method/specimen	Reference
HA is associated with malignant tumor of the pleura	Enzymatic analysis/pleural fluid	Meyer and Chaffee. 1940 [85]
HA proposed as a diagnostic tool	Electrophoresis/Pleural fluids	Boersma et al. 1975 [91]
HA as a major GAG confirms MPM diagnosis	Electrophoresis/MPM and other tumor tissues	Waxler et al. 1979 [94]
High HA is a clinical finding in MPM	GAG-degrading enzyme assay/pleural tissues	Arai et al. 1979 [36]
Increased total GAG aids in differential diagnosis between diffuse MPM and ADC	IHC/MPM tissues	Kawai et al. 1985 [95]
HA indicates diffuse MPM	Colorimetric assay/pleural fluids	Matzel and Schubert. 1979 [93]
HA aids in differential diagnosis between MPM and primary ADC	Electrophoresis/MPM and other tumor tissues	Chiu et al. 1984 [87]
Increased HA level is associated with tumor damage	IHC/pleural effusions	Thylen et al. 1999 [88] Dahl et al. 1989 [35]
HA is an indicator for MPM	HPLC/pleural and peritoneal effusions	Roboz et al. 1985 [86]
Increased serum HA indicates progressive MPM	Radiometric assay/patient serum	Frebourg et al. 1987 [34] Dahl et al. 1984 Thylen et al. 1994
HA and chondroitin sulfate are basic features of MPM	IHC/tumor tissues and pleural fluids	Nakano et al. 1986 [96]
Positive HA staining highly predictive of MPM	HA-binding probe/MPM and ADC tissues	Azumi et al. 1992 [90]
Presence of HA distinguishes MPM from ADC	IHC, HPLC/pleural effusion and tumor tissues	Klominek et al. 1989 [89] Attanoos et al. 1997 [80]
High HA with CEA aid in differential diagnosis of MPM	Radiometric assay/Pleural fluids and patient serum	Atagi et al. 1997 [92] Pettersson et al. 1988 [33]
Increased HA-binding in pleural effusion cells serves as additional diagnostic marker	[3H] hyaluronate binding assay/primary cell cultures	Teder et al. 1996 [99]
High intracellular HA delineates MPM from ADC	IHC/serous fluids	Afify et al. 2005 [81]
Increased HA level is not specific for MPM diagnosis	ELISA assay/Pleural effusions and serum	Hillerdal et al. 1991 [98]
Combination of HA and cytology may improve diagnosis of MPM	Immunoassay and IHC/pleural effusions	Welker et al. 2007 [97]
HA >100 000 ng/ml recommended as diagnostic indicator for MPM	ELISA assay pleural fluids of MPM and other tumors	Fujimoto et al. 2013 [31]
High HA is associated with MPM	IHC/MPM tissues	Töronnen et al. 2016 [83]

*MPM* malignant pleural mesothelioma, *ADC* adenocarcinoma, *HA* hyaluronic acid, *GAG* glycosaminoglycan, *IHC* immunohistochemistry, *ELISA* enzyme-linked immunosorbent assay, *HPLC* high pressure liquid chromatography

## CD44, a putative cancer stem cell marker in MPM

MPM is a notoriously chemoresistant neoplasm which led to the identification of a cancer stem cell subpopulation with the presumption that these cells are crucial candidates for conferring drug tolerance. The CSC model proposes that these cells have the capacity for self-renewal, re-initiation of tumor growth and innate resistance to chemotherapy [23, 25, 26, 100]. CD44, principally, CD44v isoforms are CSC markers that play essential roles in the execution of the fundamental features of CSCs [19, 26, 101, 102]. Hence, CD44 has been proposed to be used for the isolation and enrichment of CSCs in lung cancers including MPM [22, 24, 26, 100, 103].

That MPM contains a subpopulation of CSCs has been reported by using putative CSC markers in addition to CD44. For instance, the side population (SP) associated with CD105 [104], SP, CD9, CD24 and CD26 [105] were used in MPM cells lines and mesothelioma-derived primary cells to identify the presence of a CSC subpopulation. Using established CSC-associated genes, our group detected the presence of polycomb ring finger oncogene, (Bmi-1)^**+**^, urokinase plasminogen activator receptor (uPAR)^**+**^ and ATP-binding cassette subfamily G, member 2 (ABCG2)^**+**^ cells in 3 MPM cell lines, which elicited resistance to cisplatin and pemetrexed, indicating the presence of a drug- resistant CSC subpopulation [106]. In the H28, H2052 and Meso4 MPM cell lines, we noted a marked increase in CD44 transcript levels within the putative CSC ALDH^**+**^CD44^**+**^-sorted cells after cisplatin treatments, revealing the involvement of CD44 in ensuing drug resistance [22]. Using an activated cell sorting (FACS)-based assay, we also measured high percentages of CD44^**+**^ cells in the following MPM cell lines: H28 – 48.5%; H2052 - 57.6% and Meso4 – 50.2% relative to the entire cell population sustaining the presence of a putative CSC subpopulation (Cortes-Dericks et al., unpublished report).

There is reasonable consensus that CD44 as a putative CSC marker could identify a drug-resistant subpopulation [22, 24–26] – a possible clinical attribute of MPM, which may be considered as a new diagnostic parameter of the disease.

## “Conventional” biomarkers in pleural effusion and serum for MPM

Pleural fluid cytology is considered a reliable diagnostic tool for MPM only in experienced centres. For this reason, most of the patients undergo invasive procedures such as core-needle biopsy or video-assisted thoracoscopy to facilitate histological examination – the gold standard for MPM diagnosis [5, 107–111]. Thus, the search for a noninvasive diagnostic procedure that may confirm or exclude the diagnosis of MPM is of major clinical interest [108]. Biomarkers that can be analysed in serum, pleural effusions or blood may solve the tedious diagnostic procedure. Those that can be measured in pleural effusions have the advantage of being readily applicable for analysis at the onset of the first clinical symptoms for most patients [112] and may allow the early detection of the disease.

The “conventional” biomarkers in pleural fluid and/or serum for MPM still do not warrant an accurate diagnosis. OPN, soluble mesothelin, formerly known as soluble mesothelin-related peptide (SMRP) [113, 114], and megakaryocyte potentiating factor (MPF) also known as N-ECR/mesothelin [112, 115] are supposed to be the most promising but with some limitations due to lack of specificity and sensitivity [4, 116–118]. Mesothelin and MPF, both soluble glycoproteins [119, 120] have sufficient specificity but have a sub-optimal sensitivity for detection of MPM, being negative in both the sarcomatoid and almost half of the epitheloid subtype, particularly in the early stages [116, 121]. OPN lacks specificity for the diagnosis of MPM but may be valuable in disease monitoring [116, 122].

The glycoprotein fibulin-3 is thought to have a high diagnostic accuracy for MPM being able to distinguish between asbestos-exposed non-MPM patients and early stages of MPM [123]. However, Creany and associates [124] found that although fibulin-3 is highly expressed in MPM, its diagnostic power as a plasma or pleural effusion biomarker is less than that of mesothelin. HA as a single marker is not sufficient to discriminate MPM from benign effusion [83]. Although increased levels of HA has shown high diagnostic potential in MPM, its sensitivity and specificity is low in detecting MPM [4, 125]. Other tumor markers in serum and pleural fluid have been reported to be of diagnostic importance such as cytokeratin-19 fragment (CYFRA 21–1), carcinoembryonic antigen (CEA), cancer antigen 15–3 (CA 15–3), cancer antigen 15–9 (CA 15–9) and tissue polypeptide antigen (TPA); however, these markers are not sensitive or specific enough and, thus, cannot be applied clinically [4].

At this time, mesothelin remains the most clinically useful and the only Food Drug and Administration (FDA)-approved, single-best blood-based biomarker in the diagnosis of MPM. It has also been proposed to be a standard control in testing the sensitivity of a potential biomarker [117, 122, 124, 126–129].

## “Best practice” biomarker, mesothelin combined with other potential markers

Owing to the restrictions in sensitivity and specificity of single biomarkers, the use of combinatorial biomarkers have been evaluated to discriminate between asymptomatic asbestos-exposed subjects and early-stage MPM patients. As mesothelin is considered the most useful biomarker in MPM [117, 124, 126], a number of studies have been undertaken to enhance its diagnostic competence.

The combination of CA125 and serum mesothelin were evaluated in patients with MPM, healthy asbestos-exposed individuals, patients with asbestos-related lung disease and with benign pleural effusions to augment the sensitivity of mesothelin as a single marker. The results from this study did not show improvement of the combined CA125 and mesothelin relative to the sensitivity of mesothelin alone [130]. Notably, combined mesothelin and CEA enhanced the diagnostic accuracy in distinguishing MPM from non-small cell lung cancer (NSCLC) [131]. Muley and coworkers [132] also obtained data attesting that the dual application of mesothelin and CEA significantly increased the differential diagnosis between MPM and other lung cancers, and also between MPM and benign asbestos disease. Hence, these authors endorsed the combination of both markers for the diagnosis as well as for differential diagnosis.

The combination of serum mesothelin and HA has not improved the diagnostic performance over mesothelin alone [129]. Adversely, the team of Creany [125] furnished information that the combination of the “best practice” biomarker, mesothelin and HA have a higher diagnostic capability than using effusion mesothelin as a single marker. A comparative study between the diagnostic accuracy of fibulin-3 and mesothelin in the plasma and pleural effusions of MPM patients indicated that mesothelin generates a better diagnostic efficiency compared with fibulin-3 for MPM, whereas fibulin-3 renders superior prognostic values relative to mesothelin [124].

The combined diagnostic power of OPN and mesothelin did not provide a stronger diagnostic capacity than that of mesothelin alone [133]. Even the combination of OPN, MPF and mesothelin did not furnish a superior effect than mesothelin alone. Intriguingly, a recent study reported that the combination of serum OPN and mesothelin have a diagnostic potential in differentiating MPM from benign asbestos-related diseases and asbestos-exposed subjects [134].

At the clinical setting, studies on the combination of biomarkers have not been diagnostically satisfactory over individual markers but is encouraging as sensitive, soluble markers are emerging and being tested [9, 116, 118].

## CD44 and HA as potential biomarkers in MPM

The review of literature presented herein strongly implies that CD44 and its ligand HA are potentially useful biomarkers in MPM. The increased expression levels of both molecules in MPM tissues and pleural fluids as compared with the normal mesothelial cells strongly reinforces their implication in the development of MPM as well as their diagnostic performance. A tight CD44/HA interaction also asserts that both molecules convey biological actions in a concerted manner so that both may be considered as tandem biomarkers. CD44/HA as dual markers have been suggested in the differential diagnosis between MPM and ADC. As these markers still do not provide diagnostic accuracy, the inclusion of both CD44 and HA in a panel of biomarkers has been recommended [83]. As mentioned earlier, the combination of mesothelin/HA has shown an improved diagnostic accuracy. In this context, the incorporation of CD44 in the combined mesothelin/HA may even generate a reliable diagnosis, which warrants an extensive validation.

The proposed criteria for future biomarkers for MPM namely; their measurability in biological samples using minimally invasive tests, to differentiate MPM from benign pleural disease, applicability for all pathological subtypes and correlation with the extent of malignancy [4, 135], may be well achieved after appropriate in vitro and clinical evaluation. HA, CD44s and CD44 isoforms can readily be detected by an ELISA (enzyme-linked immunosorbent assay) assay in pleural effusion, serum, plasma and other biological fluids, which can be procured in a noninvasive procedure. Several MPM putative biomarkers have been generally based on the expression levels of molecules via mRNA microarray studies [118, 136–138], which can also be used for measuring the expression levels of CD44 and its isoforms including HA. Apart from the potential of CD44 and HA to provide an accurate diagnosis, the practicability of evaluating the expression levels also warrants faster results and less exhausting method for the patient.

## Conclusion

The general recommendations from the different pathologists worldwide for the diagnosis of MPM still advocate the use of an immunohistological examination of a conventionally stained tissue samples as the gold standard for MPM diagnosis [5, 118, 139, 140]. It still takes several months between the first signs of the disease and a definite diagnosis of MPM is achieved. Rationally, there should be a marker or a combination of markers that will offer an accurate diagnosis of MPM based on pleural effusion analysis that may be incorporated with routine immunohistochemistry or electron microscopy of cell pellets [112]. There is ample evidence that one marker alone is not sufficient to detect, differentiate and specifically diagnose MPM. Because of the tight association of HA and CD44, it stands to reason that combining the two markers or their inclusion in a panel of markers may serve as adjuvant diagnostic tool to efficiently aid the early and most likely specific diagnosis of MPM.

## Abbreviations

ABCG2: ATP-binding cassette subfamily G, member 2
ADC: Adenocarcinoma
Bmi-1: Polycomb ring finger oncogene
CA 125: Cancer antigen 125
CA 15–9: Cancer antigen 15–9
CA15-3: Cancer antigen 15–3
CEA: Carcinoembryonic antigen
CSC: Cancer stem cell
CYFRA 21–1: Cytokeratin-19 fragment
ECR/mesothelin: Precursor protein that can lead to a 31 kDa MPF or N-ECR/mesothelin-protein
EGFR: Epidermal growth factor receptor
ErbB2: Epidermal growth factor receptor-2
GAG: Glycosaminoglycan
GlcNAc: N-acetyl glucosamine
GlcUA: Glucoronic acid
GTPases: Guanosine triphosphatases
HA: Hyaluronan, hyaluronic acid
HARE: HA receptor for endocytosis
HAS: Hyaluronan synthases
HMWHA: High molecular weight hyaluronan
IHABP: Intracellular hyaluronan-binding protein
IHAPB4: Intracellular hyaluronan-binding protein 4
LMWHA: Low molecular weight hyaluronan
LYVE-1: Lymphatic vessel endocytic receptor
MAP kinase: Mitogen-activated protein kinase
MMC: Malignant mesothelioma cells
MPF: Megakaryocyte potentiating factor
MPM: Malignant pleural mesothelioma
MT1-MMP: Membrane type 1 metalloproteinase
OPN: Osteopontin
PI3/AKT: Phosphoinositide 3-kinase/protein kinase B
RHAMM: Receptor for HA-mediated motility
SMRP: Soluble mesothelin-related peptide
SP: Side population
TGFßR1: Transforming growth factor beta receptor type 1
TPA: Tissue polypeptide antigen
uPAR: Urokinase plasminogen activator receptor
